# Testicular Steroidogenesis and Locomotor Activity Are Regulated by Gonadotropin-Inhibitory Hormone in Male European Sea Bass

**DOI:** 10.1371/journal.pone.0165494

**Published:** 2016-10-27

**Authors:** José A. Paullada-Salmerón, Mairi Cowan, María Aliaga-Guerrero, José F. López-Olmeda, Evaristo L. Mañanós, Silvia Zanuy, José A. Muñoz-Cueto

**Affiliations:** 1 Department of Biology, Faculty of Marine and Environmental Sciences, University of Cádiz, Marine Campus of International Excellence (CEIMAR) and Agrifood Campus of International Excellence (ceiA3). Puerto Real, Spain; 2 INMAR-CACYTMAR Research Institutes, Puerto Real University Campus, Puerto Real, Spain; 3 Department of Physiology, Faculty of Biology, University of Murcia, Murcia, Spain; 4 Institute of Aquaculture of Torre de la Sal, CSIC, Ribera de Cabanes, Castellón, Spain; Florida International University, UNITED STATES

## Abstract

Gonadotropin-inhibitory hormone (GnIH) is a neurohormone that suppresses reproduction by acting at both the brain and pituitary levels. In addition to the brain, GnIH may also be produced in gonads and can regulate steroidogenesis and gametogenesis. However, the function of GnIH in gonadal physiology has received little attention in fish. The main objective of this study was to evaluate the effects of peripheral sbGnih-1 and sbGnih-2 implants on gonadal development and steroidogenesis during the reproductive cycle of male sea bass (*Dicentrarchus labrax*). Both Gnihs decreased testosterone (T) and 11-ketotestosterone (11-KT) plasma levels in November and December (early- and mid-spermatogenesis) but did not affect plasma levels of the progestin 17,20β-dihydroxy-4-pregnen-3-one (DHP). In February (spermiation), fish treated with sbGnih-1 and sbGnih-2 exhibited testicles with abundant type A spermatogonia and partial spermatogenesis. In addition, we determined the effects of peripheral Gnih implants on plasma follicle-stimulating hormone (Fsh) and luteinizing hormone (Lh) levels, as well as on brain and pituitary expression of the main reproductive hormone genes and their receptors during the spermiation period (February). Treatment with sbGnih-2 increased brain *gnrh2*, *gnih*, *kiss1r* and *gnihr* transcript levels. Whereas, both Gnihs decreased *lhbeta* expression and plasma Lh levels, and sbGnih-1 reduced plasmatic Fsh. Finally, through behavioral recording we showed that Gnih implanted animals exhibited a significant increase in diurnal activity from late spermatogenic to early spermiogenic stages. Our results indicate that Gnih may regulate the reproductive axis of sea bass acting not only on brain and pituitary hormones but also on gonadal physiology and behavior.

## Introduction

As in other vertebrates, reproduction in fish is controlled by a complex system of endocrine, paracrine and autocrine regulatory signals that interact along the pineal-brain-pituitary-gonadal axis [1–4]. The adequate functioning of this reproductive axis requires the synchronization of multiple hormonal systems, which is guaranteed by the presence of specific sensory organs and receptors that are able to perceive environmental, internal and social stimuli and transduce them into the secretion of neurohormones [5]. Teleost fish lack the median eminence, a vascular system characteristic of tetrapods that connects the hypothalamus with the pituitary gland. Therefore, hypothalamic neurohormones directly reach the cells of the anterior lobe of the adenohypophysis, and stimulate or inhibit the synthesis and secretion of pituitary gonadotropins, follicle-stimulating hormone (Fsh) and luteinizing hormone (Lh) that, in turn, regulate gametogenesis and gonadal steroidogenesis, as well as other processes involved in reproduction [6–10].

As in mammals, steroids play a key role in teleosts regulating gametogenesis and gonadal physiology. In male fish, the testis is the primary target organ for pituitary Fsh and Lh and, as in most vertebrates, spermatogenesis in teleosts is dependent on the action of Fsh, whereas the major role of Lh is to facilitate gamete maturation and spawning [11, 9]. Plasma levels of steroid hormones exhibit remarkable variations during male gonad maturation. Androgens such as testosterone (T) and 11-ketotestosterone (11-KT) gradually increase their levels as spermatogenesis proceeds, decreasing at spermiation. In turn, progestins such as 17α,20β-dihydroxy-4-pregnen-3-one (DHP) or 17α,20β,21-trihydroxy-4-pregnen-3-one (20ß-S) peak in the spawning season and induce spermiation, increase milt production, and stimulate spermatozoa motility [9]. Sexual steroids also play a role in transducing the sexual status to the pituitary and brain via short and long regulatory feedback, respectively. The activation of these feedback mechanisms, with both positive and negative effects, depends on the phase of the development and of the reproductive cycle [12, 3].

Since the identification of avian gonadotropin-inhibitory hormone (GNIH) [13], several studies have demonstrated that this family of neuropeptides also plays a key role in the regulation of reproduction in other vertebrates by decreasing the activity of GnRH-1 neurons and inhibiting gonadotropin synthesis and release in pituitary gonadotropes [14–18]. Nevertheless, the nature of Gnih actions in fish seems to vary depending on the species, the reproductive stage and the route of administration of this neuropeptide, and the mechanisms underlying these actions have yet to be fully elucidated. Recently, studies in teleosts such as the tilapia (*Oreochromis niloticus*) reported that the Lpxrfa-2 peptide increased the release of Lh and Fsh both *in vivo* and *in vitro* [19]. In addition, intraperitoneal administration of gfLpxrfa-3 increased pituitary *lhβ* and *fshβ* mRNA levels during early to late gonadal recrudescence in goldfish [20]. In contrast, the administration of gfLpxrfa-2 and gfLpxrfa-3 peptides suppressed *sgnrh*, *lhβ* and *fshβ* mRNA levels in the goldfish (*Carassius auratus*) [17].

Previous studies in several vertebrate species have demonstrated that GNIH and its receptor are expressed not only in the brain but also in gonads suggesting that, in addition to its influence in the synthesis and release of GNRH, LH and FSH, GNIH might regulate the reproductive axis through an autocrine/paracrine regulation of gonadal activity [21–25]. Different studies have revealed that GNIH can modulate gonadal steroidogenesis in tetrapods [21, 26–29], but evidence obtained in fish is much more scarce. Only a recent study performed in goldfish provides evidence that Gnih can be involved in male steroidogenesis, by increasing plasma T and Fsh and Lh receptor transcript levels in testicular cells [30]. The role of Gnih in the control of steroidogenesis and gametogenesis is far from being understood in this group of vertebrates.

GNIH has also been implicated in the modulation of reproductive and feeding behavior in birds and mammals [31]. It has been suggested that GNIH may inhibit male socio-sexual behavior possibly by inhibiting GNRH neurons or increasing neuroestrogen concentration by stimulating the activity of brain cytochrome P450 aromatase. Moreover, GNIH stimulates feeding behavior by modulating the activities of hypothalamic and central amygdala neurons and the release of different orexigenic and anorexigenic neuropeptides [31]. In fish, terminal nerve Gnrh-3 neurons show spontaneous pacemaker activity and appear responsible for controlling the motivational or arousal state of the animal, including sexual behavior [32–33]. Although RFRP-2 cannot be considered a true ortholog of Gnih, like RFRP-1 and RFRP-3, it has been shown recently that its synaptic release from hypothalamic neurons may inhibit the pacemaker activity of these GnRH-3 neurons by modulating the opening and closing of ionic channels, and acting as a negative motivational signal for sexual behavior [33].

The European sea bass (*Dicentrachus labrax*) is an important teleost species for marine aquaculture in Europe but still presents problems under farming conditions related to reproduction, such as early puberty and unbalanced sex proportions [34]. This species has also represented an interesting fish model for the study of environmental and endocrine control of reproduction [18, 34–45]. Recently, we identified a *gnih* gene in sea bass encoding a prepro-*gnih* mRNA that gives rise to two different RFamide peptides, named as sbGnih-1 and sbGnih-2, which are produced by proteolytic processing from a single protein precursor [45]. In a previous study [18], we reported an inhibitory role of Gnih (mainly sbGnih-2) in the reproductive axis of sea bass by acting at the brain and pituitary level. The intracerebroventricular administration of Gnih down-regulated the brain expression of several genes of the Gnrh and kisspeptin systems, as well as pituitary gonadotropins mRNA levels and plasmatic Lh in sea bass [18]. In the present study, we aimed to evaluate whether Gnih may also play a role in the regulation of steroidogenesis and gonadal maturation in the European sea bass. For this purpose, we performed peripheral implants of sbGnih during critical periods of the reproductive cycle covering pre-spermatogenesis, spermatogenesis and spermiation stages (from October to February), and analyzed its effects on plasma steroids and gonadotropins, as well as on the expression of different brain and pituitary reproductive hormone genes. Finally, as the European sea bass has been reported to exhibit a phase inversion of its diel feeding and locomotor activity pattern, i.e., nocturnal in winter coinciding with the spawning period and diurnal during the remainder of the year [46, 47], we decided to also analyze the effects of Gnih on locomotor activity in this species.

## Materials and Methods

### Animals

Twenty month-old specimens of European sea bass (body length and weight of 21± 0.1cm and 123.13 ± 3 g, respectively, at the beginning of the experiment), were obtained from CUPIMAR S.L (San Fernando, Spain) and housed under natural conditions of photoperiod and temperature, at a salinity of 39 ppt, in the “Laboratorio de Cultivos Marinos” (University of Cádiz, Puerto Real, Spain, 36° 31' 51.55" N, 6° 12' 38.78" W). Animals were fed one time per day (9.30 h local time) with a commercial diet using automatic feeders (1% of body weight, L6 Obtibass Skretting España S.A, Burgos Spain). All animals were treated in agreement with the European Union Regulation (EC. Directive 86/609/EEC) concerning the protection of experimental animals. Animal experimental protocols were approved by the Animal Care and Use Committee of the University of Cádiz. Measures were taken to avoid the suffering of the animals.

### Hormones

Based on available sea bass gonadotropin-inhibitory precursor sequence [GenBank accession no. LN681205, 45], sea bass sbGnih-1 (PLHLHANMPMRF-NH_2_) and sbGnih-2 (SPNSTPNMPQRF-NH_2_) peptides were synthesized by Thermo Fisher Scientific GmbH (Ulm, Germany). Synthetic peptides were amidated at the C terminal end and purified by high-performance liquid chromatography (>95% purity). The peptides were dissolved in coconut oil, which was also used as a vehicle in control animals. The coconut oil was melted in a glass vial at 35°C, and then mixed with sbGnih-1 and sbGnih-2 peptides and placed in an ultrasonic bath to facilitate dissolution. This preparation was injected as a warm fluid that solidifies within the fish to produce a slow releasing sbGnih emulsion.

### Experimental procedure and sampling

In October 2014, fifty-one specimens of European sea bass were distributed randomly among three tanks. Seventeen fish were assigned to each of three treatment groups: sbGnih-1, sbGnih-2, and controls, which only received coconut oil. All the fish were subjected to the same routine and all procedures were carried out between 9:00 and 11:00 h. At the starting point of the experiment (October 17), all fish were anesthetized and weighed. Control fish were injected with 160 μl of coconut oil alone; fish from the second group received a dose of 1μg sbGnih-1/g of body weight (bw) in 160 μl of coconut oil; animals from the third group were injected with a dose of 1μg sbGnih-2/g bw in 160 μl of coconut oil. Each dose was injected intramuscularly one time per month, from October to January (day 17 of each month). Injections were done alternately on the left or right sides of the fish body each month, at its rostral-dorsal pole. In all groups, blood samples were collected 5 days post injection (day 22 of each month) from the caudal vessels using heparinized syringes and expelled into cold heparinized tubes. At the end of the experiment (February 17, 2015), all fish were anesthetized by immersion in MS-222 and sacrificed, and the brain and pituitary gland were immediately dissected, frozen in liquid nitrogen and stored at -80°C for subsequent RNA extraction. Blood samples were centrifuged at 3000 rpm at 4°C for 15 min and the plasma stored at -20°C until analysis. The gonads were removed and a small sample preserved in formalin for histological studies. Female specimens were not considered for the analysis.

### RNA extraction and reverse transcription for real-time quantitative PCR analysis (qPCR)

Total RNA from sea bass brain and pituitary was extracted with TRIsure reagent (Bioline, London, UK), according to the manufacturer’s protocol. Tissues were homogenized in a mixer mill MM400 (Retsch, Haan, Germany) using 4–5 stainless steel spheres. Total RNA concentration was quantified on a NanoDrop 2000 spectrophotometer (Thermo Fisher Scientific). Total RNA (1μg) was retro-transcribed and DNA removed using a Primer Scrip^TM^ RT Reagent Kit with gDNA Eraser (TAKARA BIO. INC, Japan). Primers for qPCR assay and amplicon sizes are shown in Table 1. Real time PCR analysis was performed in a PCR Bio-Rad CFX96 Touch detection system (Bio-Rad, Richmond, CA), using the SYBR Premix ExTaq^TM^ (Tli RNase H Plus; TAKARA BIO. INC, Japan) as reported previously [18]. Briefly, PCR conditions were as follow: initial denaturation 2 min at 95°C and 40 cycles of 15 sec at 95°C sec at the optimal temperature from each primers pairs (detailed in Table 1) for annealing-extension. Duplicates of each sample were analyzed in the same test.

**Table 1:** Primers used for quantitative real-time PCR.

Gene	GenBank Accesion no.	5’ to 3’ sequence	Annealing temperature (°C)	Amplicon size (bp)
*gnrh1*	AF224279	fw: GGTCCTATGGACTGAGTCCAGG	61	131
		rv: TGATTCCTCTGCACAACCTAA		
*gnrh2*	AF224281	fw: GTGTGAGGCAGGAGAATGCA	61	81
		rv: CTGGCTAAGGCATCCAGAATG		
*gnrh3*	AF224280	fw: TGTGGGAGAGCTAGAGGCAAC	60	81
		rv: GTTTGGGCACTCGCCTCTT		
*gnrhr-II-2b*	AJ606685	fw: AGACTCTGAAGATGACGGTGGT	60	250
		rv: AGTGAAGCGTCTCTTCTCATCC		
*gnrhr-II-1a*	AJ419594	fw: CTCTGGCTATCAATAAGGC	60	125
		rv: CTCGGGATGGATGATGGT		
*kiss1*	FJ008914	fw: GCATCAATACTGGCATCAGCAAAGA	63	94
		rv: TCAACCATTCTGACCTGGGAAACTT		
*kiss2*	FJ008915	fw: GGGAGGATTCCAGCCCGTGTTTCT	61	104
		rv: GAGGCCGAACGGGTTGAAGTTGAA		
*kiss1r*	JN202446	fw: TGGTGGCTCTGTTCCTCATCT	63	78
		rv: CGTAACTGCGTAGGCCAAAAG		
*kiss2r*	JN202447	fw: CGTCACAGTCTACCCCCTGAA	63	69
		rv: CAGATGCTGACAATCATGGCTACT		
*lhβ*	AF543315	fw: TTGAGCTTCCTGACTGTCCA	60	177
		rv: GCAGGCTCTCGAAGGTACAG		
*fshβ*	AF543314	fw: ACCAACATCAGCATCCAAGTG	63	127
		rv: TTCTCTGTTCAGGCCTCTCATAGT		
*sbgnih*	LN681205	fw: CCCACCACCAGCAAAATCAGCC	61	176
		rv: TCCCAAGACCTTCCGAACCTC		
*sbgnihr*	LN681208	fw: GTACGGAAGCATCGGAGTCAAAC	60	178
		rv: CCAGGACAGCATGAAAAGCAAAG		
*l17*	AF139590	fw: CAGGAGTGGGTGACATGGTC	60,5	97
		rv: GACTTCCGCTGCCGTATCAC		
*18s*	AY831388	fw: TCAGACCCAAAACCCATGCG	60	182
		rv: ACCCTGATTCCCCGTTACCC		
*elfα*	AJ866727	fw: CTGTGCTGATCGTTGCTGCTGGTGTT	61	75
		rv: CGTGCTCGCGGGTCTGTCC		

Standard curves were generated for each gene with 10-fold serial dilutions of cDNA and all calibration curves exhibited slopes close to -3.32 and efficiencies around 100%. Melting curves were performed for each sample in order to confirm that a single product was amplified. The expression of the target genes was normalized against three different reference genes (*18s*, *l17 and elf*α). The relative expression of genes analysed was calculated by the ΔΔ-Ct method [48].

### Hormone Analysis

Plasma levels of T and 11KT were measured by specific ELISAs as described previously [49, 50]. Plasma concentrations of DHP were measured by specific ELISAs based on the method described by Nash [51]. Plasma levels of Lh and Fsh were determined using two respective homologous ELISAs developed for sea bass [52, 53].

### Histological analysis

Testis were removed, weighed and fixed in 4% paraformaldehyde solution in 0,1M phosphate buffer, pH 7.4, for 48 hours at 4°C. Then, samples were dehydrated in a graded series of ethanol, embedded in paraffin and 7 μm-thick sections were sectioned in a microtome followed by hematoxylin and eosin (HE) staining. The phases of testicular gametogenesis and the associated stages of testicular development were identified by light microscopy following the criteria established previously by Espigares et al. [54] and Begtashi et al. [55], respectively: immature testes, stage I; proliferative, stage II; meiotic/early spermiogenic, stages III–IV and spermiating, stage V.

### Activity Monitoring

Tanks were each fitted with a photoswitch (model E3S-AD62, Omron, Japan) at the bottom of the tank (30 cm below the surface and 8 cm from the bottom). Photoswitches were connected to a computer and worked by emitting a continuous infrared light beam. Interruptions in the beam caused by fish movements within 20 cm were recorded on the computer and organised into 10 min bins using specialised software (DIO96USB, University of Murcia, Spain). Locomotor activity was continuously recorded for the whole duration of experiments.

### Statistical Analyses

The results are presented as the mean ± SEM. Locomotor activity records and diurnalism were analyzed using the software package for chronobiological studies (El Temps v 1.291, Prof. A. Díez-Noguera, University of Barcelona) and Microsoft Excel^®^. Statistical differences in gene expression, hormonal levels and diurnalism of locomotor activity were determined by ANOVA followed by Student-Newman-Keuls post hoc test, using Statgraphic Plus 5.1 software (Statpoint Technologies, Warrenton, VA, USA). Before the analysis, data were checked for normality and homogeneity of variance, and the values were log-or square root- transformed when required. When the data did not fulfill the requirements of the ANOVA, data were analyzed using the non-parametric Kruskal-Wallis ANOVA on ranks followed by Bonferroni’s test. Statistical significance was established as P<0.05. All graphics were created using PRISM 6 software.

## Results

### Effects of sea bass Gnih on plasma sex steroid levels and gonadal development

**Fig 1:**
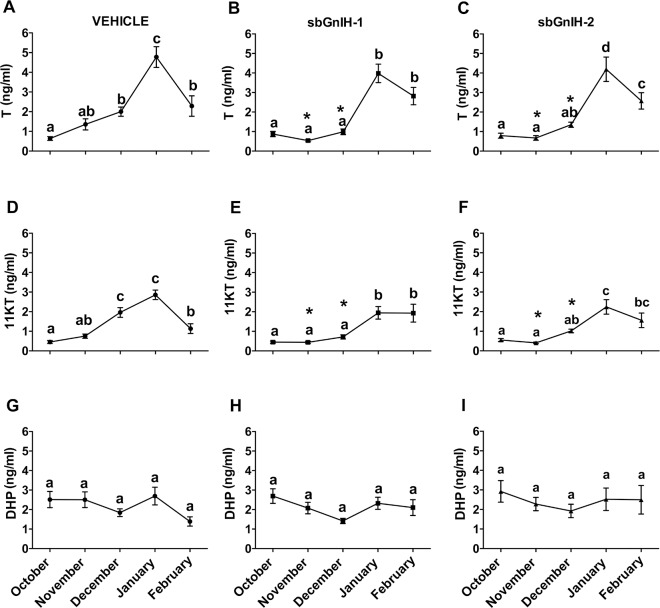
*In vivo* effect of sbGnih-1 and sbGnih-2 in male sea bass steroid plasma levels. Plasma levels of testosterone (T)(A, B, C), 11-ketotestosterone (11-KT)(D, E, F) and 17α,20β-dihydroxy-4-pregnen-3-one (DHP)(G, H, I) were analyzed in sbGnih-1- (B, E, H) and sbGnih-2- (C, F, I) treated animals throughout the reproductive cycle. Vehicle implanted animals represent the control group (A, D, G). Values are expressed as mean ± SEM (n = 12–14). Different lower case letters indicate significant differences throughout the reproductive cycle within each condition (ANOVA, Student-Newman Keuls test, P<0.05). Asterisks (*) denote significant differences between Gnih-treated animals *versus* controls in a particular month (P<0.05).

Control animals exhibited similar plasma profiles of testosterone T and 11-KT along the 5-month experimental period, with lowest levels in October and November, a significant increase in December and January, when both androgens peaked, decreasing thereafter in February (Fig 1A and 1D). Although comparable reproductive profiles of plasma T and 11-KT were also observed in Gnih-implanted animals, both sbGnih-1 and sbGnih-2 significantly decreased T (Fig 1B and 1C) and 11-KT (Fig 1E and 1F) plasma levels in November and December (P<0.05). In contrast, plasma levels of DHP did not show apparent variations along the reproductive cycle nor significant effects of sbGnih (Fig 1G, 1H and 1I).

**Fig 2:**
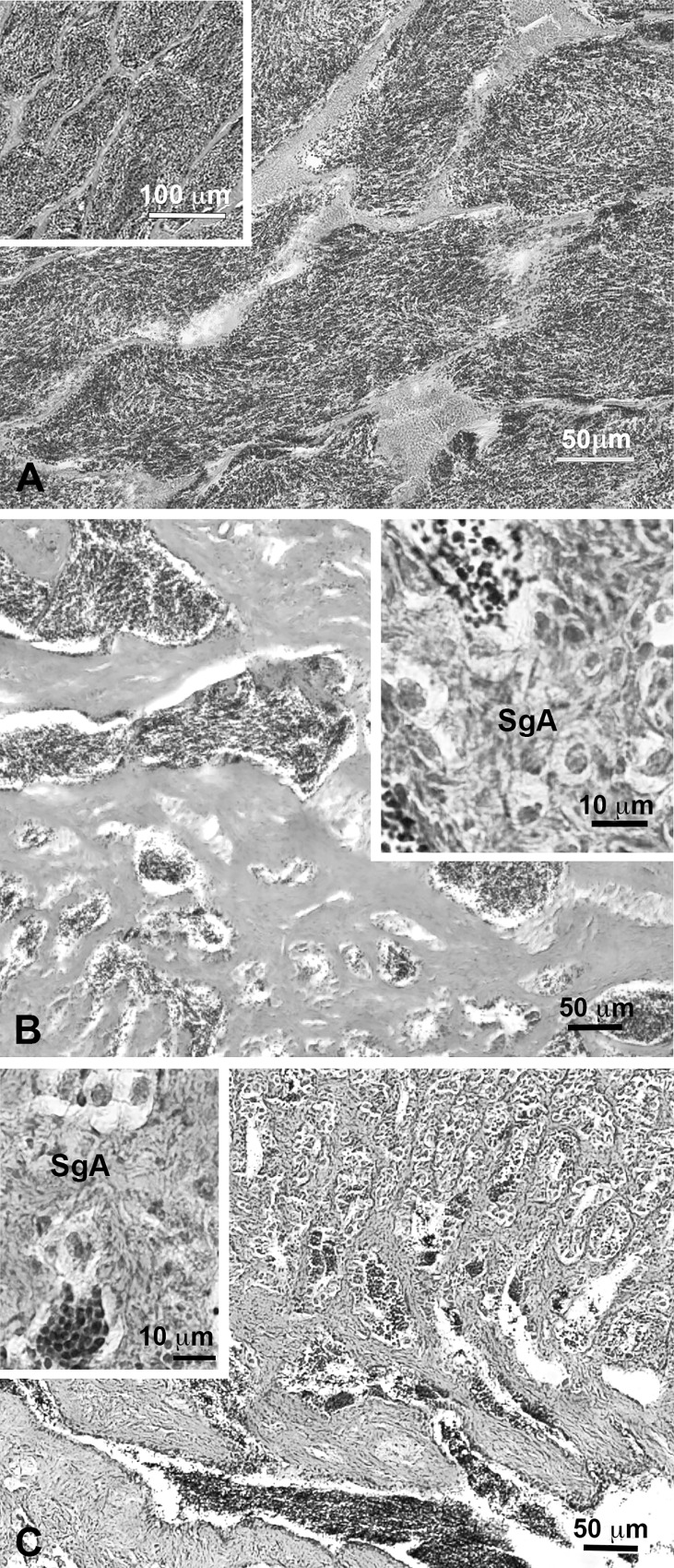
Histological analysis of testicles of European sea bass at end of the experiment (February, spermiation). Full spermiating control fish (A) with lobules mostly filled with sperm (inset). Gnih-1 (B) and Gnih-2 (C) treated fish testicles exhibiting only isolated clusters of sperm and abundant type A spermatogonia (SgA, insets). Bars 50 μm, 100 μm (inset in A) and 10 μm (insets in B and C).

Although no significant differences in gonadosomatic indexes were observed (1.69 ± 0.23; 1.47 ± 0.25 and 1.74 ± 0,23 for controls, sbGnih-1 and sbGnih-2 implanted animals, respectively), histological analysis revealed marked differences in testicles from controls and Gnih implanted fish (Fig 2, Table 2). In control fish only late meiotic and full spermiogenic testicles containing mostly sperm and some cysts of spermatocytes and spermatids were observed (Fig 2A, Table 2). However, both sbGnih-1 and sbGnih-2 treated fish also exhibited a significant percentage of testicles containing abundant type A spermatogonia (SgA) and scattered isolated clusters of spermatozoids (Fig 2B and 2C, Table 2), which resulted in a partial spermatogenesis instead of complete, as observed in controls.

**Table 2:** Developmental phases of testicular gametogenesis (%) exhibited by controls and sbGnih-implanted fish at the end of the experiment (February).

Developmental phase	Controls (n = 12)	sbGnih-1 (n = 14)	sbGnih-2 (n = 14)
Late meiotic/Early spermiogenic (IV)	33.3	28.6	42.85
Spermiogenic (V)	66.7	57.1	42.85
Partial spermatogenesis*	0	14.3	14.3

(*) Partial spermatogenesis refers to testicles with scattered clusters of spermatozoa and spermatogonia.

### Effects of sea bass Gnih on the expression of brain reproductive hormone genes

**Fig 3:**
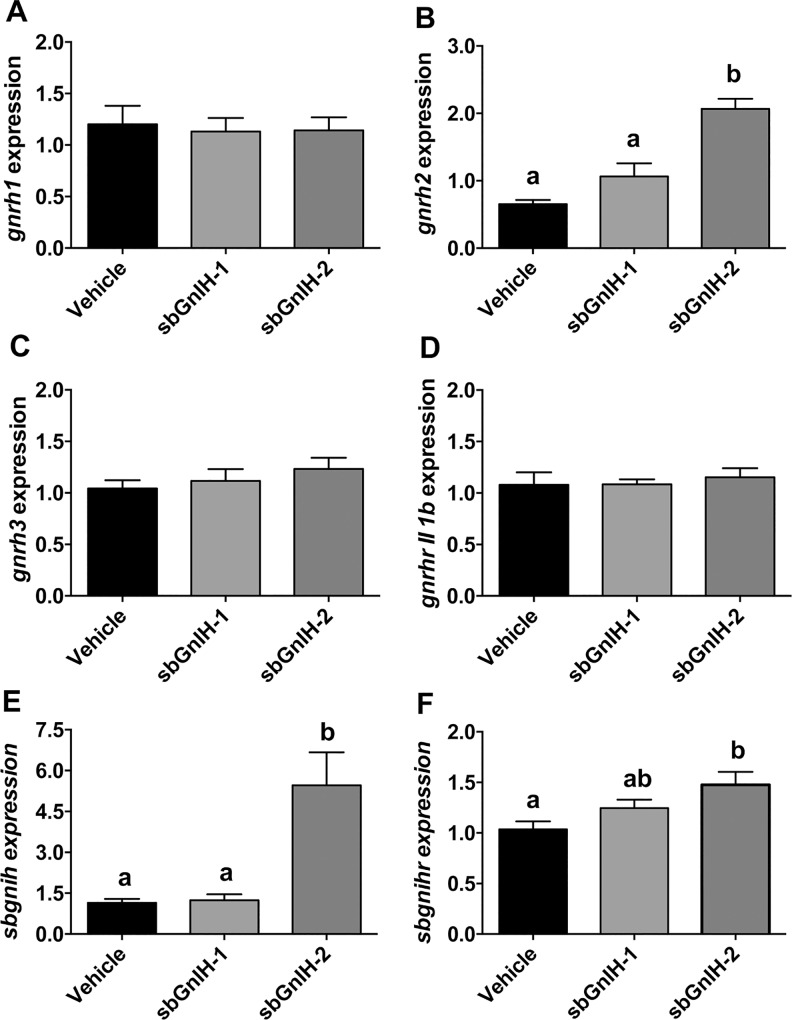
Effect of *in vivo* implants of sbGnih-1 and sbGnih-2 on brain expression of reproductive hormone genes. Data show transcript levels of *gnrh1* (A), *gnrh2* (B), *gnrh3* (C), *gnrhr-II-2a* (D), *sbgnih* (E) and *sbgnihr* (F) in male sea bass specimens at the spermiation stage (February). Vehicle implanted animals represent the control group. Values are expressed as mean ± SEM (n = 12–14). Different lower case letters indicate significant differences between treatments (ANOVA, Student-Newman Keuls test, P<0.05).

**Fig 4:**
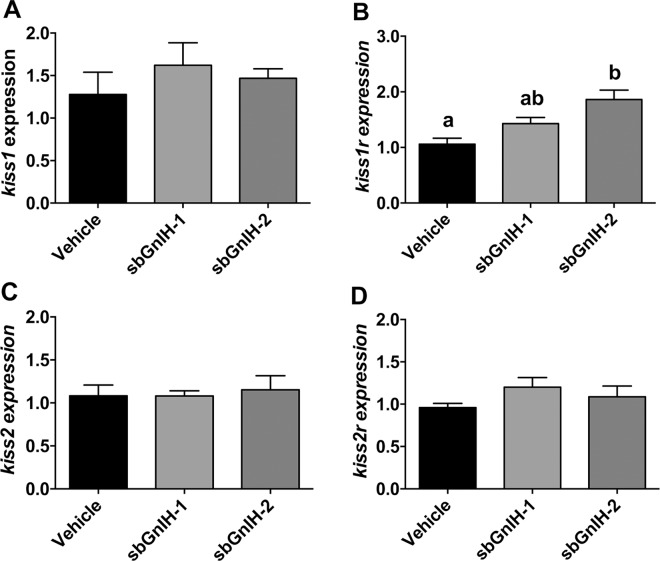
Effect of *in vivo* implants of sbGnih-1 and sbGnih-2 on brain expression of reproductive hormone genes. Data show transcript levels of *kiss1* (A), *kiss1r* (B), *kiss2* (C) and *kiss2r* (D) in male sea bass specimens at the spermiation stage (February). Vehicle implanted animals represent the control group. Values are expressed as mean ± SEM (n = 12–14). Different lower case letters indicate significant differences between treatments (ANOVA, Student-Newman Keuls test, P<0.05).

We also analyzed the effects of Gnih on transcript levels of different genes of the Gnrh (*gnrh1*, *gnrh2*, *gnrh3*, *gnrhr-II-2b*), kisspeptin (*kiss1*, *kiss2*, *kiss1r*, *kiss2r*) and Gnih (*sbgnih*, *sbgnihr*) systems. Implants of sbGnih-2 increased *gnrh2* (Fig 3B), *sbgnih* (Fig 3E), *sbgnihr* (Fig 3F) and *kiss1r* (Fig 4B) mRNA levels. No significant effect of sbGnih-2 was observed in the expression of the other genes analyzed (Fig 3A, 3C and 3D and Fig 4A, 4C and 4D). None of the brain reproductive genes studied elicited significant changes in their expression in sbGnih-1 treated animals (Figs 3 and 4).

### Effect of sea bass Gnih on pituitary gene expression and plasma gonadotropins levels

**Fig 5:**
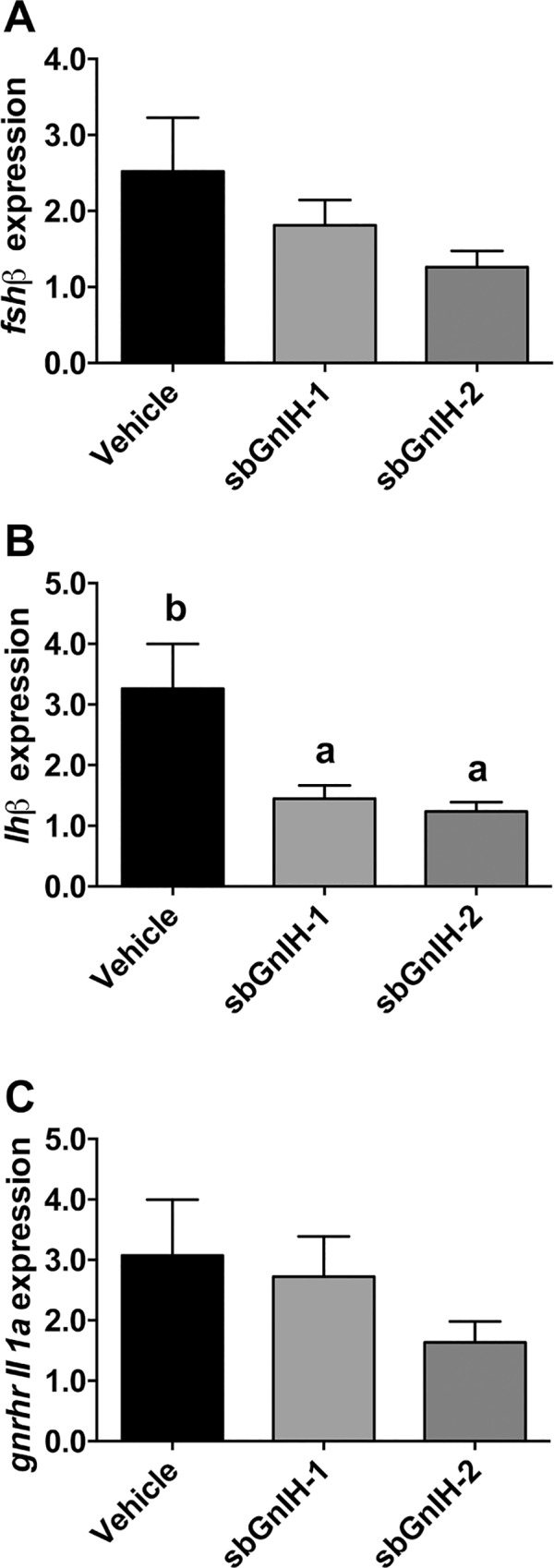
Effect of *in vivo* implants of sbGnih-1 and sbGnih-2 on pituitary expression of reproductive hormone genes. Data show transcript levels of *fshβ* (A), *lhβ* (B) and *gnrhr-II-1a* (C) in male sea bass specimens at the spermiation stage (February). Vehicle implanted animals represent the control group. Values are expressed as mean ± SEM (n = 12–14). Different lower case letters indicate significant differences between treatments (ANOVA, Student-Newman Keuls test, P<0.05).

**Fig 6:**
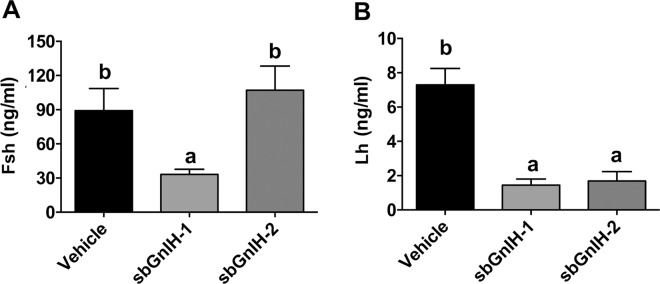
Effect of *in vivo* implants of sbGnih-1 and sbGnih-2 on plasma levels of Fsh and Lh. A, plasma levels of Fsh and B, plasma levels of Lh in male sea bass specimens at the spermiation stage (February). Vehicle implanted animals represent the control group. Values are expressed as mean ± SEM (n = 12–14). Different lower case letters indicate significant differences between treatments (ANOVA, Student-Newman Keuls test, P<0.05).

Fish implanted with both sbGnih-1 and sbGnih-2 exhibited reduced *lhβ* subunit mRNA levels in the pituitary of sea bass (Fig 5B). In contrast, pituitary *fshβ* mRNA content and *gnrhr-II-1a* transcript levels were not significantly affected by either of the Gnih treatments (Fig 5A and 5C). We also analyzed the effect of sbGnih-1 and sbGnih-2 implants on plasma Fsh and Lh levels at the spermiation stage (February). A significant decrease in plasma Fsh levels was observed in animals treated with sbGnih-1 but not in sbGnih-2 implanted animals (Fig 6A). Both sbGnih-1 and sbGnih-2 significantly reduced plasma Lh levels in male sea bass (Fig 6B).

### Effects of sea bass Gnih on locomotor activity

**Fig 7:**
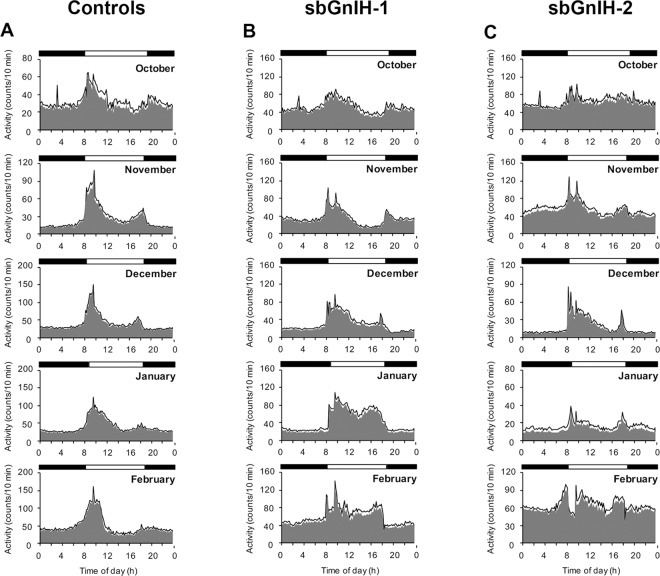
Effect of sbGnih-1 and sbGnih-2 implants on locomotor activity of male sea bass. Average diel profiles of locomotor activity (mean waveforms) of controls (A), sbGnih-1- (B) and sbGnih-2- (C) implanted fish from October to February. Each point in the mean waveform was calculated as mean ± SEM from the 10-min binned data over the days of the respective month, indicated at the top right of the mean waveform. The grey area in the waveform represents the mean values and the continuous line the SEM. Horizontal bars above graphs indicate day-time (open bars) and night-time (solid bars).

The representative actograms and mean waveforms of locomotor activity of controls, sbGnih-1 and sbGnih-2 implanted animals are presented in S1 Fig and Fig 7. The average total daily activity tended to be higher in fish implanted with sbGnih-1 (4913 ± 130, 5398 ± 201 and 4999 ± 321 counts/day for controls, sbGnih-1 and sbGnih-2 implanted animals, respectively), although no statistically significant differences were observed (Kruskal-Wallis, p > 0.05). The analysis of actograms and mean waveforms revealed that in controls this activity was concentrated at the beginning of the day, with a secondary peak of activity at the end of the diurnal phase (S1A Fig and Fig 7A). In sbGnih-1 treated animals, locomotor activity extended throughout almost the entire light phase from December to February (S1B Fig and Fig 7B), whereas a remarkable increase in diurnal activity was observed in sbGnih-2 treated animals in December (S1C Fig and Fig 7C).

**Fig 8:**
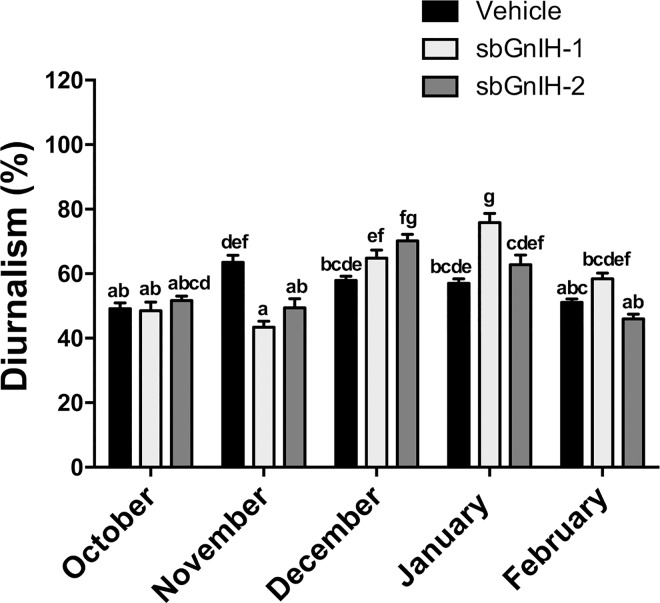
Effect of sbGnih-1- and sbGnih-2-implants on average monthly percentage of diurnal locomotor activity in male sea bass. Values are expressed as mean ± SEM from October to February. Different lower case letters indicate significant differences between treatments (Kruskal-Wallis ANOVA on ranks followed by Bonferroni’s test, P<0.0001).

The quantification of diurnal locomotor activity per month revealed significant differences between groups (Kruskal-Wallis, p<0.0001). In controls, the percentage of activity during the light phase increased from October to November (from 49.2 ± 1.8% to 63.6 ± 2.1%), decreasing significantly from November to February up to 51.1 ± 1.1% (Fig 8). In contrast, both sbGnih-1 and sbGnih-2 implanted animals exhibited a markedly different pattern of locomotor activity as the reproductive cycle progressed, with sustained percentage of diurnal activity from October to November (from 43.4 ± 1.9% to 51.7 ± 1.4%), a significant increase in their diurnalism in December and January (up to 70.2 ± 2% and 75.9 ± 2.8% for sbGnih-2 and Gnih-1, respectively), and a reduction in their activity during the light phase in February (between 46.0 ± 1.4% and 58.4 ± 1.8%), when Gnih treatment ceased (Fig 8).

## Discussion

Since its identification in the year 2000, GNIH has been clearly established as a negative regulator of reproduction in birds and mammals by acting mainly at the brain and pituitary levels [13, 56–60]. On the other hand, several studies have also reported that GNIH and/or its receptor (GNIHR) are expressed in gonads [21–24, 28] and are involved in the regulation of steroidogenesis in avian and mammalian species [23, 24, 26, 29]. In fish, the mechanisms of actions of Gnih on gonadotropin release and the reproductive axis are not so clear and contradictory findings have been reported [16, 18, 19, 45, 61, 62]. In addition, Gnih functions and implications in steroidogenesis and gametogenesis in teleosts have not been sufficiently assessed. In order to fill this gap, in the present study we performed a long term experiment aiming at identifying the actions of Gnih in steroidogenesis and gametogenesis in the testis of the European sea bass.

The present study provides evidence for the inhibitory effects of Gnih on steroid secretion and testicular development in the teleost fish European sea bass. The profiles of T and 11-KT reported are in accordance with previous results obtained in sea bass [49, 54], with levels increasing during mid-late recrudescence (December and January), and falling once spermiation begins (February). In fish, T levels increase in both male and female during gonadal development, while 11-KT is considered to play an important role in stimulating spermatogenesis in the testis of several fish species, including sea bass [49, 63, 64]. Our findings revealed that prolonged sbGnih-1 and sbGnih-2 treatments during the reproductive cycle inhibited the testicular steroidogenesis (T and 11-KT but not DHP secretion) in particular reproductive stages (early- to mid-spermatogenesis, November and December), and that these effects were accompanied by evidence of partial and delayed spermatogenesis instead of complete, as observed in controls, as well as by a decrease in gonadotropin synthesis and release in the spermiation period (February). Taken together with data obtained in a previous study performed in our laboratory, also in sea bass [18], the present results reinforce the assumption that Gnih is able to act at all the levels of the brain-pituitary-gonad axis controlling reproduction in this species. Similarly, previous studies in birds and mammals have also reported a decrease in sex steroid levels after GNIH treatment. In male quail, continuous peripheral administration of GNIH suppressed plasma T [21]. RFRF-3 treatment also caused inhibitory effects on T synthesis in mice testis [29], as well as on the synthesis and release of T in male pig [65]. In the only study performed in a fish species, the administration of gfLpxrfa enhanced serum T levels, increased the expression of StAR and 3β-hydroxysteroid dehydrogenase (3β-HSD) mRNAs and decreased the expression of cytochrome P450 aromatase gene in male goldfish [30]. In contrast, no apparent seasonal variations nor effects of sbGnih on DHP were observed. The plasma levels of DHP, a possible maturation inducing steroid in European sea bass [66], were neither greatly affected by Gnrha treatments in this species [67]. It is possible that Gnih could have effects on DHP within the testes, which is not reflected by changes in plasma concentration.

It is well-known that testicular activity of vertebrates, including fish, is under the control of the Fsh and Lh [6, 7, 9]. In the present study, we have shown that sbGnih-1 and/or sbGnih-2 are able to reduce the expression of *lhβ* subunit and plasma levels of Lh and Fsh. Therefore, the inhibitory effect of Gnih on the steroidogenesis and the spermatogenic cycle might be due to the suppressive effects of Gnih on gonadotropin synthesis and/or release in sea bass. Moreover, a direct effect of Gnih in sea bass testis cannot be discarded because increasing evidence suggests that Gnih and its receptor are expressed in the gonads. In this sense, *GNIHR* mRNA has been detected in starling testis [21], in myoid cells of rodent testis [24, 28] and in the interstitial testicular tissue of goldfish [30]. In sea bass testis, both *gnih* mRNAs [45], and *gnihr* transcript levels (unpublished data) are present. Further *ex vivo* and *in vitro* studies could contribute to clarify the nature (direct *versus* indirect) of the actions of Gnih in gonadal physiology.

In the present work, we have shown that only peripheral sbGnih-2 implant was able to elicit significant effects (stimulation) on *gnrh2*, *gnih*, *gnihr* and *kiss1r* gene expression in the sea bass brain, reinforcing the conclusion obtained in a previous study [18] that considers sbGnih-2 as the main functional peptide of this neuroendocrine system in the reproductive axis of sea bass and the only one that exerts an autocrine regulation in the brain Gnih system of this species. But in contrast to the present results, Paullada [18] showed that central injection of sbGnih-2, significantly decreased *gnrh2*, *gnih*, *gnihr* and *kiss1r* mRNA levels in the brain of sea bass at 6 hours post injection. It should be noted that different routes of administration of Gnih peptides (intracerebroventricular *versus* peripheral implants) and elapsed times after treatments (short term *versus* long term) were used in both studies, which could explain the apparent disagreement in results. Studies performed in fish have revealed contradictory effects of Gnih, the nature of these effects (stimulatory or inhibitory) varying considerably depending on the species, the physiological status, the route of administration of the Gnih peptide or the time elapsed between treatment and analysis [17–20, 61, 68]. Sexual steroids play a key role in referring the reproductive status to the brain and pituitary and their precise mode of action is not completely understood in fish [69]. Interestingly, mammalian GNIH neurons express steroid receptors (ERα and AR), which are responsible for direct steroid response [57, 70]. Therefore, we cannot exclude the possibility that the effects of sbGnih-2 on brain *gnrh2*, *gnih*, *gnihr* and *kiss1r* transcript levels resulted from the sex steroids feedback, as a consequence of the observed Gnih effect on androgen (T and 11-KT) levels in the sea bass. However, this hypothesis does not seem plausible because both sbGnih-1 and sbGnih-2 decrease T and 11-KT plasma levels in sea bass but only sbGnih-2 stimulated brain *gnrh2*, *gnih*, *gnihr* and *kiss1r* gene expression.

Our data show that *lhβ* gene expression in February (spermiation stage) was significantly lower in fish treated with both sbGnih-1 and sbGnih-2 compared to the control group. According to these effects, the animals treated with both peptides also exhibited a significant decrease in plasma levels of Lh. These results are in agreement with those obtained in our previous study, in which central injection of sbGnih-2 reduced *lhβ* gene expression, and both peptides demonstrated their effectiveness in reducing Lh plasma levels [18]. On the other hand, the animals treated with sbGnih-1 also showed a significant decrease in Fsh plasma levels compared to the control group, which was not evident in our former study [18]. Gnih inhibits Lh and Fsh release in intact pituitary cultures of the cichlid fish *Cichlasoma dimerus* [62] and Gnih-2 peptide also decreased *lhβ* mRNA levels in the pituitary gland of the grouper *Epinephelus coioides* [71]. In goldfish, Gnih-2 peptide administered intraperitoneally decreased both *lhβ* and *fshβ* mRNA levels and in zebrafish (*Danio rerio*) Lpxrfa3 also reduced plasma Lh in this species [16, 17]. However, inhibitory or stimulatory effects of Gnih on Fsh and Lh plasma levels were observed in the goldfish at different gonadal stages [20, 61]. In contrast to our results, Lpxrfa peptides increased the *in vitro* release of Lh and Fsh in tilapia [19] and sockeye salmon *Oncorhynchus nerka* [68], as well as the expression of gonadotropins in grass puffer *Takifugu niphobles* [72]. Again, whether these discrepancies of Gnih actions in fish pituitary reflect real differences among species, or are dependent on the type of study (*in vivo* or *in vitro*), the reproductive stage of the animals, the route of administration of the peptide and/or the time elapsed until between treatments and analysis remain to be deciphered.

A direct action of Gnih in the pituitary of sea bass is suggested by the presence of sbGnih-immunoreactive fibers in the proximal pars distalis, in close proximity to Fsh and Lh cells [45]. But it must be taken into account that sex steroids can also act on gonadotropin cells of the fish pituitary and, therefore, modulate pituitary functions through an indirect regulatory feedback mechanism, which can be inhibitory or stimulatory depending on the phase of the reproductive cycle [3]. The classical inhibitory actions of gonadal steroids on gonadotropin secretion at the end of the reproductive cycle have been demonstrated by using gonadectomy and treatment with T and/or estradiol (E2) in a variety of teleost species, including sea bass [73–76]. In the present study we reported that T and 11-KT plasma levels decreased from January to February (late spermatogenesis to spermiation) in control sea bass, but a significant decrease of both androgens was not evident in sbGnih-1 treated animals, nor for 11-KT in sbGnih-2 treated fish. Thus, it is possible that the inhibition of both sbGnih-1 and sbGnih-2 peptides on Fsh and Lh plasma levels could be mediated by the negative feedback of these androgens on the sea bass pituitary.

The European sea bass exhibits diurnal feeding and locomotor activity patterns during the resting season, switching to nocturnal in winter coinciding with the spawning period [46, 47]. The behavioral analysis performed in this study showed that both Gnihs affected the diurnal to nocturnal ratio of locomotor activity along the reproductive cycle. Control animals progressively increase their nocturnal habits from early spermatogenesis (November) to spermiation (February), while Gnih implanted animals exhibited a significant increase in diurnal activity from late spermatogenic (December) to early spermiogenic (January) stages, increasing the nocturnalism only when the Gnih treatment ceased (February). GNIH has been found to be involved in the modulation of socio-sexual behavior in birds by acting on GNRH-2 cells and promoting the conversion of T into neurostrogens via the stimulation of brain cytochrome P450 aromatase activity [31]. Interestingly, we have shown that both Gnih forms decreased T plasma levels and sbGnih-2 increased brain *gnrh2* gene expression in sea bass. Whether these effects are involved in the behavioral actions of Gnih in locomotor activity reported in the present study requires further investigation. Research in progress in our laboratory is being directed towards elucidating whether Gnih modulates the activity of aromatase and other enzymes involved in the synthesis of neurosteroids in the sea bass brain.

In summary, in the present study we have reported that, in addition to its brain and pituitary actions, Gnih can affect the reproductive axis of sea bass by modulating gonadal physiology and behavior. We have shown that Gnih effects on testicular steroidogenesis are restricted to particular stages of the reproductive cycle (early-, mid-spermatogenesis) and steroids (androgens, not progestins) but determined marked effects on the progression of gametogenesis, as revealed in the histological analysis at the end of the study (February, spermiation period). In agreement with our previous study [18], Gnih inhibits gonadotropin synthesis and release, in particular Lh. Also consistent with our former results, sbGnih-2 appears to be the most active form of Gnih in sea bass, at least in the brain. The apparent controversy observed in the nature of Gnih effects on the brain of sea bass between the present study (stimulatory) and our previous study (inhibitory) reflects that the mode of administration (central or peripheral, single or repeated injections), and the elapsed time between treatment and analysis (short term or long term) must be taken into account to avoid confusion. Finally, we have provided evidence, for the first time in fish, that Gnih can modulate the behavior in this group of vertebrates. Considering that *gnih* expression decreases from resting to the spawning season, when sea bass adopts its nocturnal habits, and that Gnih increases the diurnal activity of this species as the reproductive cycle progresses, we can hypothesize that Gnih could represent a neuroendocrine signal for diurnalism in this species.

## Supporting Information

S1 Fig Effect of sbGnih-1 and sbGnih-2 implants on locomotor activity of male sea bass. Locomotor actograms representing the recording of group activity of controls (A), sbGnih-1- (B) and sbGnih-2- (C) implanted fish. In the actograms, data have been double plotted (48-h scale) for convenient visualization. Activity was binned every 10 min and the height of each point represents the number of interruptions of the infrared light beam. Horizontal bars above graphs indicate day-time (open bars) and night-time (solid bars).
(TIF)
