# Supplementary figures and images for: Testicular Steroidogenesis and Locomotor Activity Are Regulated by Gonadotropin-Inhibitory Hormone in Male European Sea Bass
Source: PLoS One. 2016 Oct 27;11(10):e0165494. doi: 10.1371/journal.pone.0165494 (PMC5082886; doi:10.1371/journal.pone.0165494)

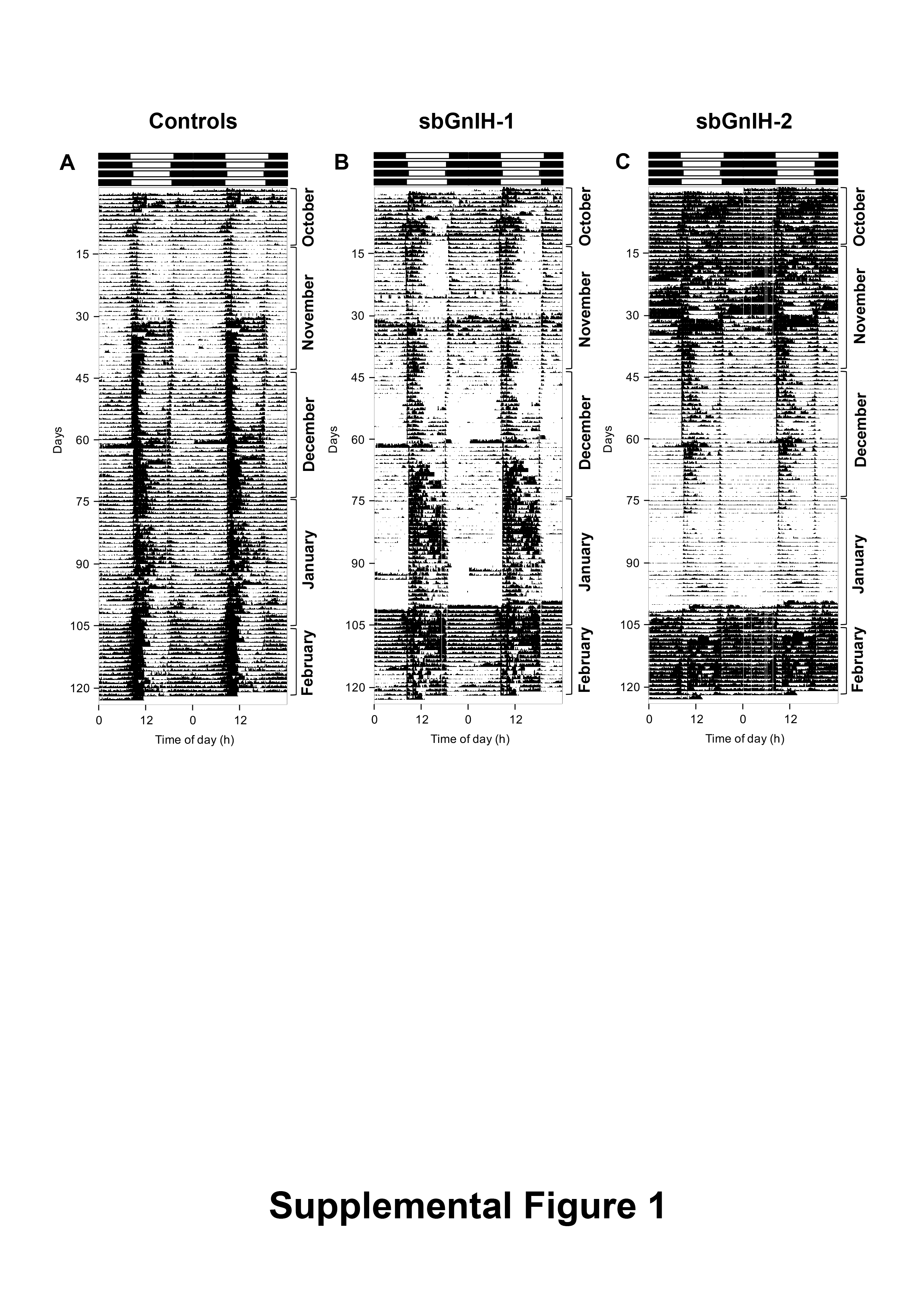

Supplement: S1 Fig — Locomotor actograms representing the recording of group activity of controls (A), sbGnih-1- (B) and sbGnih-2- (C) implanted fish. In the actograms, data have been double plotted (48-h scale) for convenient visualization. Activity was binned every 10 min and the height of each point represents the number of interruptions of the infrared light beam. Horizontal bars above graphs indicate day-time (open bars) and night-time (solid bars). (TIF) [file pone.0165494.s001.tif]
